# Non-alcoholic fatty liver disease is not a causal risk factor for psoriasis: A Mendelian randomization study of 108,835 individuals

**DOI:** 10.3389/fimmu.2022.1022460

**Published:** 2022-10-24

**Authors:** Charlotte Näslund-Koch, Stig Egil Bojesen, Lise Lotte Gluud, Lone Skov, Signe Vedel-Krogh

**Affiliations:** ^1^ Department of Dermatology and Allergy, Copenhagen University Hospital - Herlev and Gentofte, Copenhagen, Denmark; ^2^ Department of Clinical Medicine, University of Copenhagen, Copenhagen, Denmark; ^3^ Department of Clinical Biochemistry, Copenhagen University Hospital - Herlev and Gentofte, Copenhagen, Denmark; ^4^ Copenhagen General Population Study, Copenhagen University Hospital - Herlev and Gentofte, Copenhagen, Denmark; ^5^ Gastro Unit, Copenhagen University Hospital–Hvidovre, Copenhagen, Denmark

**Keywords:** psoriasis, non-alcoholic fatty liver disease (NAFLD), causality, Mendelian Randomization (MR), observational analyses, genetic analyses, epidemiology

## Abstract

**Background:**

Psoriasis is observationally associated with a higher risk of non-alcoholic fatty liver disease (NAFLD); however, the causal relationship between the two diseases remains unclear.

**Objective:**

We hypothesized that individuals with NAFLD or elevated liver fat content have higher risk of psoriasis and that NAFLD is a causal risk factor for psoriasis. We tested this using a Mendelian randomization approach.

**Methods:**

We included 108,835 individuals from the Danish general population, including 1,277 individuals with psoriasis and 802 individuals with NAFLD according to ICD codes. To estimate liver fat content, a subset of the participants (N = 7,416) also had a CT scan performed. First, we tested whether a diagnosis of NAFLD or elevated liver fat content was observationally associated with risk of psoriasis. Subsequently, we used the genetic variants *PNPLA3* and *TM6SF2*, both strongly associated with NAFLD and high liver fat content, to test whether NAFLD was causally associated with increased risk of psoriasis.

**Results:**

Observationally, individuals with vs. without a diagnosis of NAFLD had higher risk of psoriasis with an odds ratio of 2.03 (95% confidence interval 1.28-3.21). The risk of psoriasis increased in a stepwise manner with increasing liver fat content with an odds ratio of 5.00 (2.63-9.46) in individuals in the highest quartile of liver fat content compared to individuals in the lowest quartile. In genetic analyses, *PNPLA3* and *TM6SF2* were both associated with increased risk of NAFLD but not with increased risk of psoriasis.

**Conclusion:**

Observationally, a diagnosis of NAFLD or elevated liver fat content was associated with higher risk of psoriasis. However, using genetic variants as a proxy for NAFLD, we did not find evidence of a causal relationship between NAFLD and psoriasis. Thus, the observational association between NAFLD and psoriasis is presumably a result of shared confounding factors or reverse causation.

## Introduction

Psoriasis is a chronic inflammatory immune-mediated disease once considered only a skin disease but today acknowledged as a systemic inflammatory disease. Psoriasis is associated with extensive comorbidities such as obesity, the metabolic syndrome, type 2 diabetes, cardiovascular disease, and non-alcoholic fatty liver disease (NAFLD) (1, 2). NAFLD, which has a prevalence of 25% worldwide (3), ranges from non-alcoholic fatty liver (NAFL) characterized by hepatic steatosis (>5% of fat content in the liver) to non-alcoholic steatohepatitis (NASH) characterized by steatosis, inflammation, and varying degrees of fibrosis (4). Patients with NAFLD, particularly those with NASH, have increased liver-specific and cardiovascular morbidity and mortality (5). During the last decade, interest in NAFLD among patients with psoriasis has increased as several observational studies of psoriatic patients have reported higher risk of NAFLD (6, 7) even after adjusting for confounders such as obesity and the metabolic syndrome (8–10). The risk of psoriasis in patients with NAFLD is less studied; however, circulating pro-inflammatory cytokines associated with both development of psoriasis and NAFLD could drive a chronic low-grade inflammation potentially linking the two diseases (11, 12). Yet, as confounders and reverse causation are inherent limitations in observational studies the causal relationship between NAFLD and psoriasis remains unclear. Using the epidemiological approach called Mendelian randomization (MR), genetic variants can be used to investigate causal associations in an observational setting (13). Here, genetic variants are used as robust proxies for modifiable exposures and as germline genetic variants are randomly distributed at conception, and present at birth, they are not associated with confounders nor subject to reverse causation. Two genetic variants are strongly associated with liver fat content and NAFLD: a common variant in the gene encoding the protein patatin-like phospholipase domain containing 3 protein (*PNPLA3*) and a variant in the transmembrane 6 superfamily member 2 (*TM6SF2*) (14, 15). Using these genetic variants as proxies for high liver fat content and NAFLD, we tested the hypothesis that genetically determined high liver fat content is a causal risk factor for psoriasis in a MR study of 108,835 individuals from the general population.

## Materials and methods

### Study participants

The Copenhagen General Population Study (CGPS) (16) is a cohort study of the general population in Denmark. Individuals aged 20-100 and living in greater Copenhagen were randomly recruited from 2003-2015. Participation rate was 43%. At examination, participants filled out a questionnaire, underwent a physical examination, and had blood sample drawn for biochemical and genetic analyses. A random subset of the participants was also offered a computed tomography (CT) scan at the time of enrollment. The detailed scanning protocol have previously been published (17). To avoid population stratification (13), we only included individuals of Danish descent in this study.

All participants gave written informed consent. The study was conducted according to the Declaration of Helsinki and approved by Danish ethical committees (H-KF-01-144/01).

### Study design

Observational and genetic data were from the same individuals. First, we did observational analyses to estimate the risk of psoriasis among participants with a diagnosis of NAFLD or elevated liver fat content. Second, we did a one-sample one-directional MR study, using genetic variants as proxies for NAFLD and high liver fat content. A genetic variant is considered a valid instrument in a MR-study if certain assumptions are met: 1) it should be robustly associated with the exposure of interest (NAFLD and elevated liver fat content) 2) it should be independent of confounding factors that could affect the exposure (NAFLD) or the outcome (psoriasis) 3) it should not be associated with the outcome (psoriasis) expect *via* the exposure (NAFLD) (13).

### Hospital-diagnosed psoriasis

In Denmark, all individuals receive a unique personal identification number which can be linked to nationwide health registers (18). Using this, we linked individuals from CGPS to the national Danish Patient Registry which contains diagnoses according to the World Health Organization International Classification of Diseases (WHO ICD) codes for all in- and outpatients in Danish hospitals since 1977 (19). Individuals with psoriasis were identified by hospitalization (in- or outpatient) using ICD-8 696.09, 696.10, 696.19, and ICD-10 L40, corresponding to moderate to severe psoriasis (20). From January 1977 to December 2018, 1,277 individuals were diagnosed with psoriasis. In sensitivity analyses, we used a more restrictive definition of psoriasis using only ICD-8 code 696.19 and ICD-10 codes L40.0 and L40.9.

### Non-alcoholic fatty liver disease and liver fat content

NAFLD was identified using the ICD-10 codes K75.9, K76.0 and K76.9, as done previously (21). In sensitivity analyses, we used a more restrictive definition of NAFLD using only ICD-8 code 571.11 and ICD-10 code K76.0.

A diagnosis of NAFLD may include diagnostic imaging (e.g., ultrasound, transient elastography, magnetic resonance elastography, or CT) (22, 23), combined with exclusion of other hepatic disease and/or daily alcohol consumption of ≥30 g for men and ≥20 g for women. However, the NASH diagnosis requires a liver biopsy (4). Unfortunately, we did not have access to histological information. From January 1977 to December 2018, 802 individuals were diagnosed with NAFLD.

In a subset of participants (N = 7,416) liver fat content was estimated by liver attenuation on CT-scans measured in Hounsfield units (HU), which is a measure of the radiodensity of a substance on a CT scan. Normal liver density is 60 ± 6 HU. Hounsfield units of the liver inversely correlates with liver fat content, and a threshold of 48 HU has previously been shown to be 100% specific for moderate to severe steatosis (24). To provide a more understandable measurement of liver fat content, we converted Hounsfield units to *percent liver fat content*, as previously done (21, 25).

In a sensitivity analysis, we used the fatty liver index (FLI), a non-invasive biomarker that can be used to predict fatty liver in the general population (26), as a proxy for NAFLD. We used information on gamma-glutamyl transferase (GGT), triglycerides (TG), body mass index (BMI), and waist circumference (WC) measured at baseline and calculated FLI using the following algorithm: FLI=((exp(0.953*log_e_(TRIG)+0.139*(BMI)+0.718*log_e_(GGT)+0.053*(WC)-15.745))/(1+exp(0.953*log_e_(TRIG)+0.139*(BMI)+0.718*log_e_(GGT)+0.053*(WC)-15.745)))*100. A fatty liver index <30 can be used to exclude fatty liver disease (26).

### Genotypes

Genotyping of *PNPLA3* I148M (rs738409) and *TM6SF2* E167K (rs58542926) was done using TaqMan based Assays (Applied Biosystems, Foster City, CA, USA) or by an allele-specific PCR system (KASP genotyping technology; LGC Genomics, Hoddesdon, Herts, UK) (27). We combined the two genotypes to an *allele count* according to the total number of NAFLD-promoting alleles for each participant (0-4). Due to few individuals with 4 NAFLD-promoting alleles, we combined individuals with 3 or 4 alleles into one group.

### Covariates

Body mass index (BMI) was calculated from measured weight (kg) divided by measured height squared [m (2)]. Obesity was defined as BMI ≥30 kg/m (2). Hypertension was defined as systolic blood pressure ≥140 mm Hg, and/or diastolic blood pressure ≥90 mm Hg, or self-reported use of antihypertensive treatment. Dyslipidaemia was defined as non-fasting low-density lipoprotein (LDL) cholesterol ≥3 mmol/L (115 mg/dL), and/or total-cholesterol ≥5 mmol/L (190 mg/dL), and/or self-reported use of statins. Remnant cholesterol was calculated as total-cholesterol – LDL cholesterol – HDL cholesterol. Low physical activity was defined as less than 2 hours of light physical activity in leisure time per week. Type 2 diabetes mellitus was defined as self-reported diabetes, self-reported use of anti-diabetic medication, non-fasting plasma glucose > 11 mmol/L (198 mg/dL), or as a registered diabetes diagnose prior to examination obtained from the national Danish Patient Registry using World Health Organization (WHO) International Classification of Diseases (ICD) codes (ICD-8 250 and ICD-10 E11, E13, and E14). The systemic immune-inflammation index (blood neutrophil count x blood platelet count/blood lymphocyte count) was included as a marker of inflammatory and immune status. A cut-off value of 575.8 have previously been shown to be associated with psoriasis activation (28). Plasma levels of non-fasting triglycerides were measured using standard hospital assays and hypertriglyceridemia was defined as triglycerides ≥2 mmol/L (175 mg/dL) (29). Alcohol consumption was self-reported and excessive alcohol consumption was defined as alcohol consumption >84 g/week for women and >168 g/week for men.

### Statistical analysis

We used Stata/SE 17.0 (StataCorp, College Station, Texas). A two-sided P-value less than 0.05 was considered statistically significant. A Chi-squared test was used to evaluate Hardy–Weinberg equilibrium. For comparisons of baseline characteristics between individuals with and without NAFLD, we used Wilcoxon rank-sum test for continuous variables and Pearson’s x (2) test or Kruskall-Wallis test by ranks for categorical variables. Observational association between NAFLD, liver fat content, and psoriasis was assessed using logistic regression. Risk of psoriasis as a function of liver fat content on continuous scales was assessed using restricted cubic splines (30). We used three knots chosen according to Akaike´s information criterion and parsimony to secure best fit of the model. Participants with >30% liver fat content (N=16) were excluded from the analyses to avoid bias of the estimates due to instability of outlier values. Risk of NAFLD and psoriasis according to genotype and total allele count was assessed using logistic regression. As genotypes are present at birth and thus not prone to confounding, analyses were only adjusted for age and sex, on which we had full information in all individuals.

In sensitivity analyses, observational association between NAFLD and psoriasis was assessed using logistic regression stratified according to potential confounders. A likelihood ratio test was used to test for interaction between the included confounders and NAFLD on the risk of psoriasis.

## Results

In total, we included 108,835 individuals with information on NAFLD and psoriasis in the observational analyses. Characteristics of the participants at the day of examination are shown in **Supplementary Table S1**. Individuals with NAFLD were slightly older, more likely to be women, had a higher BMI and waist circumference as well as higher levels of triglycerides and non-fasting glucose compared to individuals without NAFLD. A larger proportion of individuals with NAFLD had hypertension, type 2 diabetes, and low level of physical activity. Finally, individuals with NAFLD were more likely to be smokers and had a slightly lower alcohol consumption.

Hardy-Weinberg equilibrium did not show sign of genotyping or population sampling errors (*P*-value for *PNPLA3* = 0.51, *P*-value for *TM6SF2* = 0.48).

### Observational analyses

**Figure 1:**
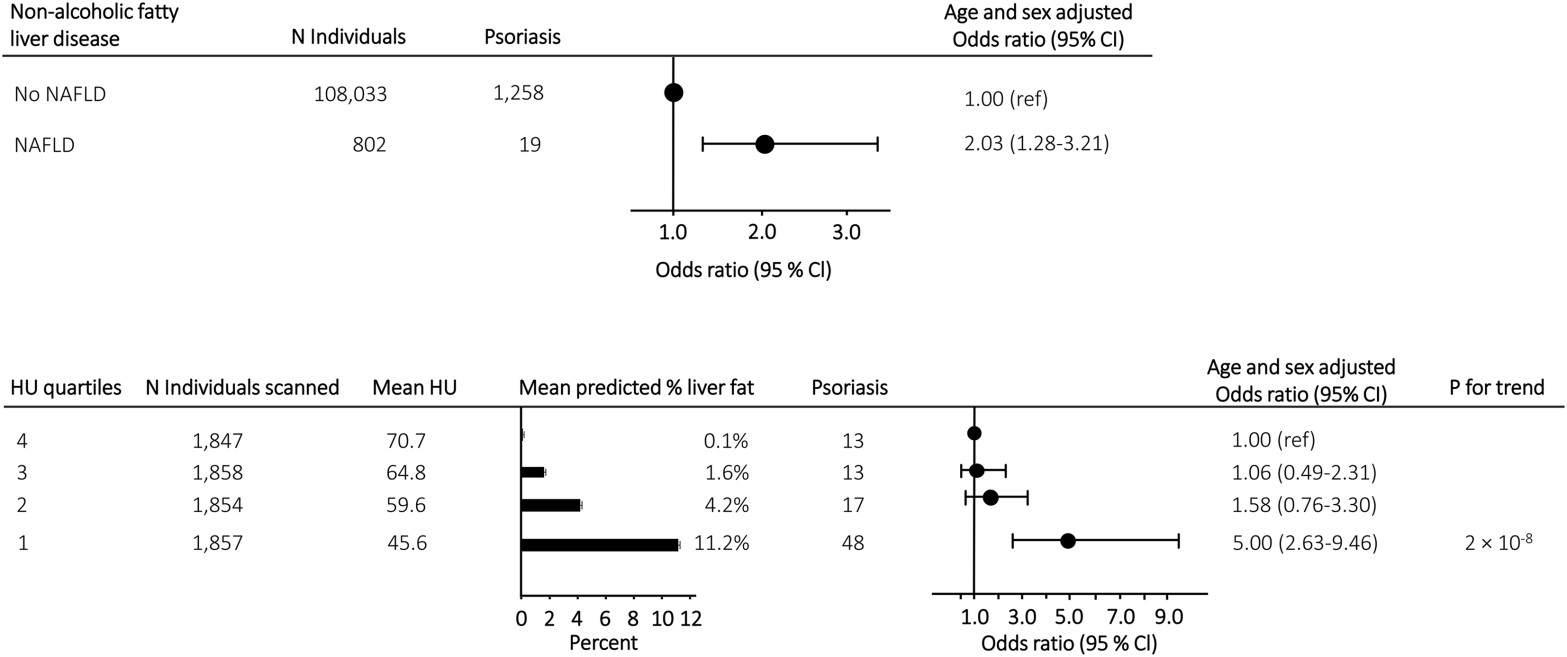
Upper panel: Risk of psoriasis in individuals with NAFLD compared to individuals without NAFLD from the general population. Lower panel: Mean predicted liver fat content and risk of psoriasis in 7,416 individuals with information on liver fat content from CT-scans. Individuals were grouped according to liver fat content in quartiles and risk assessment was done with individuals in the lowest quartile (lowest liver fat content) as reference. Analyses were adjusted for sex and age. NAFLD, non-alcoholic fatty liver disease; N, number; HU, Hounsfield units.

**Figure 2:**
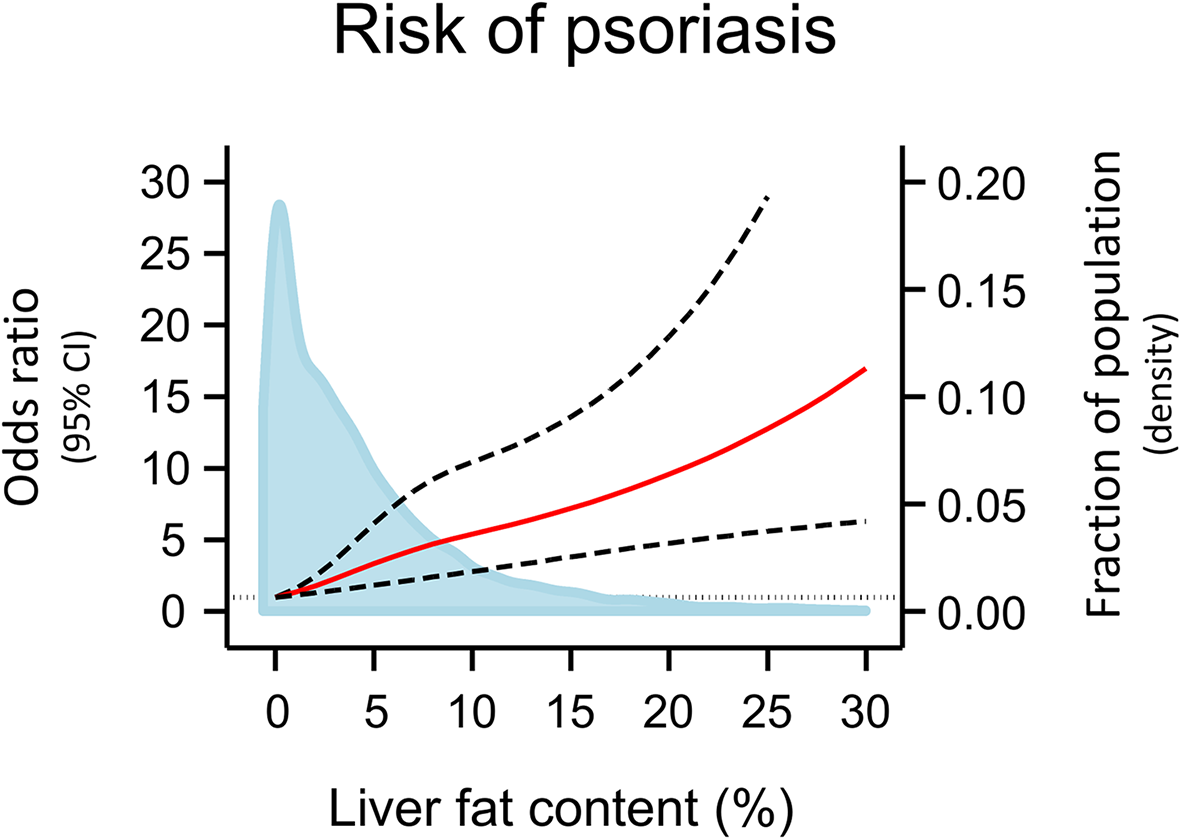
Risk of psoriasis according to liver fat content on continuous scales using data from the 7,416 individuals with information on liver fat content. Odds ratios (red line) and 95% confidence intervals (dashed lines) are from logistic regression using restricted cubic splines, adjusted for age and sex. The grey vertical dashed line corresponds to odds ratio = 1.00. Individuals with 0% liver fat content (N=1,522) was used as reference. Individuals with >30% liver fat content (N=16) were excluded from the analyses to avoid bias of the estimates due to instability of outlier values. Density plot of liver fat content in the population distribution (blue) are made as kernel density estimation. CI, Confidence interval.

Of the 108,835 participants included in this study, 802 had NAFLD and 1,277 had psoriasis. The age and sex adjusted odds ratio of psoriasis was 2.03 (95% confidence interval 1.28-3.21) in individuals with vs. without NAFLD (**Figure 1** upper panel). Among the 7,416 participants who underwent a CT scan, the risk of psoriasis increased in a stepwise manner with increasing liver fat content (P for trend 2 x 10^-8^) and an odds ratio of 5.00 (2.63-9.46) in individuals in the highest quartile of liver fat content compared to individuals in the lowest quartile (**Figure 1** lower panel). Similar results were seen when adjusting the association of NAFLD, liver fat content and psoriasis for triglycerides, dyslipidaemia, and remnant cholesterol measured at baseline **(**compare **Figure 1** with **Supplementary Figure S1** **)**, when using the restrictive diagnostic definitions of NAFLD and psoriasis (compare **Figure 1** with **Supplementary Figure S2**), and when using the fatty liver index calculated at baseline visit as a marker of NAFLD (compare **Figure 1** with **Supplementary Figure S3**). On continuous scales using restricted cubic splines, the risk of psoriasis increased with increasing liver fat content (**Figure 2**).

The higher risk of psoriasis among individuals with NAFLD was consistent in sensitivity analyses stratified according to potential confounders including sex, obesity, systemic immune-inflammation index, triglycerides, and type 2 diabetes mellitus (**Supplementary Figure S4**). We found no interaction between the included confounders and NAFLD on the risk of psoriasis. When further assessing the association between liver fat content and psoriasis, we found an age and sex adjusted odds ratio of 1.19 (1.09-1.29) per doubling in liver fat content (**Supplementary Figure S5**). In analyses stratified according to sex, obesity, systemic immune-inflammation index, triglycerides, and type 2 diabetes mellitus, we found no interaction between potential confounders and doubling of liver fat content on the risk of psoriasis.

### Genetic analyses

**Figure 3:**
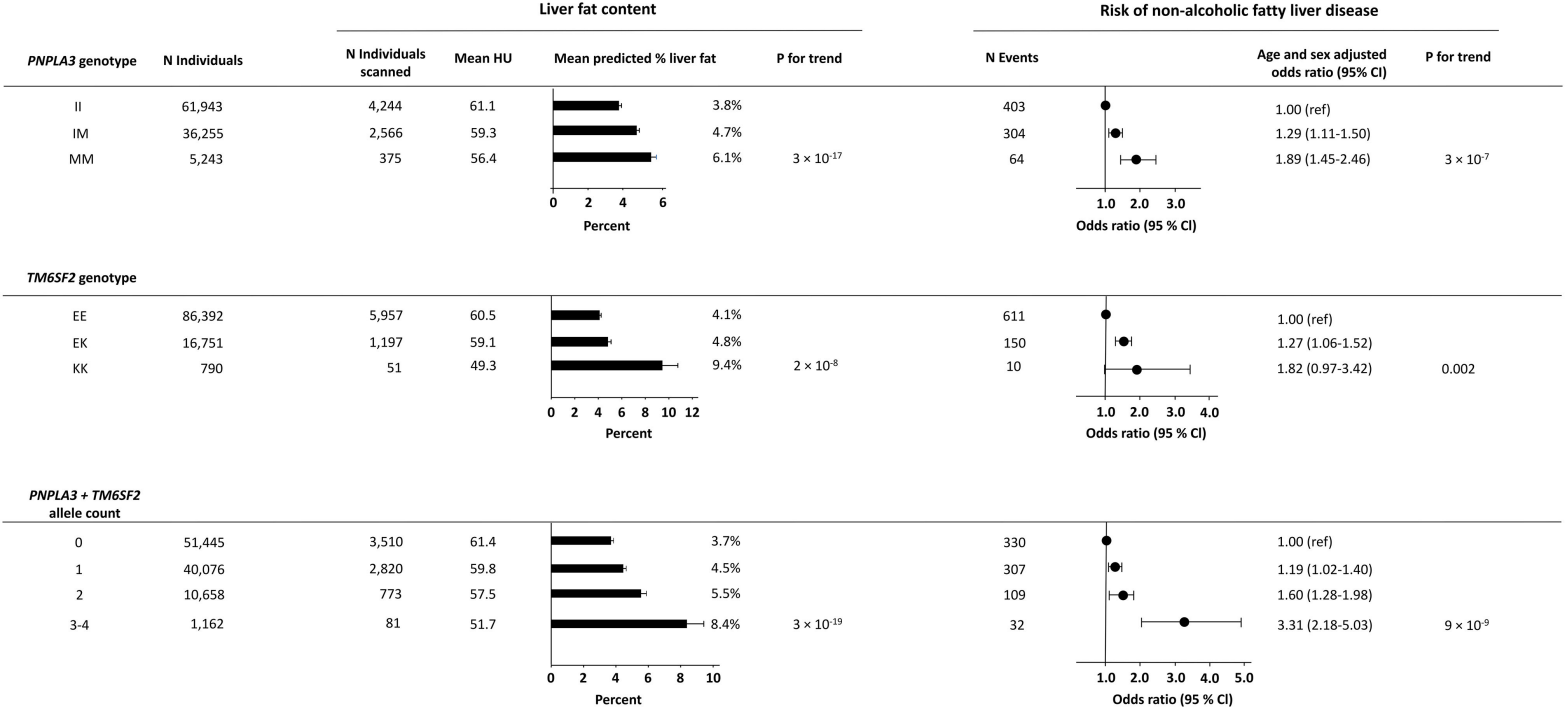
Liver fat content according to NAFLD-promoting genetic variants in 7,205 individuals and risk of NAFLD according to NAFLD-promoting genetic variants in all individuals. Upper panel: Liver fat content and risk of NAFLD according to the genotype *PNPLA3*. Middle panel: Liver fat content and risk of NAFLD according to the genotype *TM6SF2*. Lower panel: Liver fat content and risk of NAFLD according to the combined allele count. Analyses were adjusted for age and sex. N, number; NAFLD, non-alcoholic fatty liver disease; HU, Hounsfield units.

Among 103,441 individuals included in the genetic analyses of *PNPLA3* rs738409, 61,943 were non-carriers, 36,255 were heterozygotes, and 5,243 were homozygotes for the M-allele. Among 103,933 individuals included in the genetic analyses of *TM6SF2* rs58542926, 86,392 were non-carriers, 16,751 were heterozygotes, and 790 were homozygotes for the K-allele. In combined analyses of the genetic variants, 103,341 individuals with information on both *PNPLA3* and *TM6SF2* were included. As expected, a stepwise increase in liver fat content and risk of NAFLD was seen across genotypes for both *PNPLA3*, *TM6SF2*, and combined allele counts (**Figure 3**). In sensitivity analyses, results were similar when using a more restrictive definition of NAFLD (compare **Figure 3** with **Supplementary Figure S6**).

**Figure 4:**
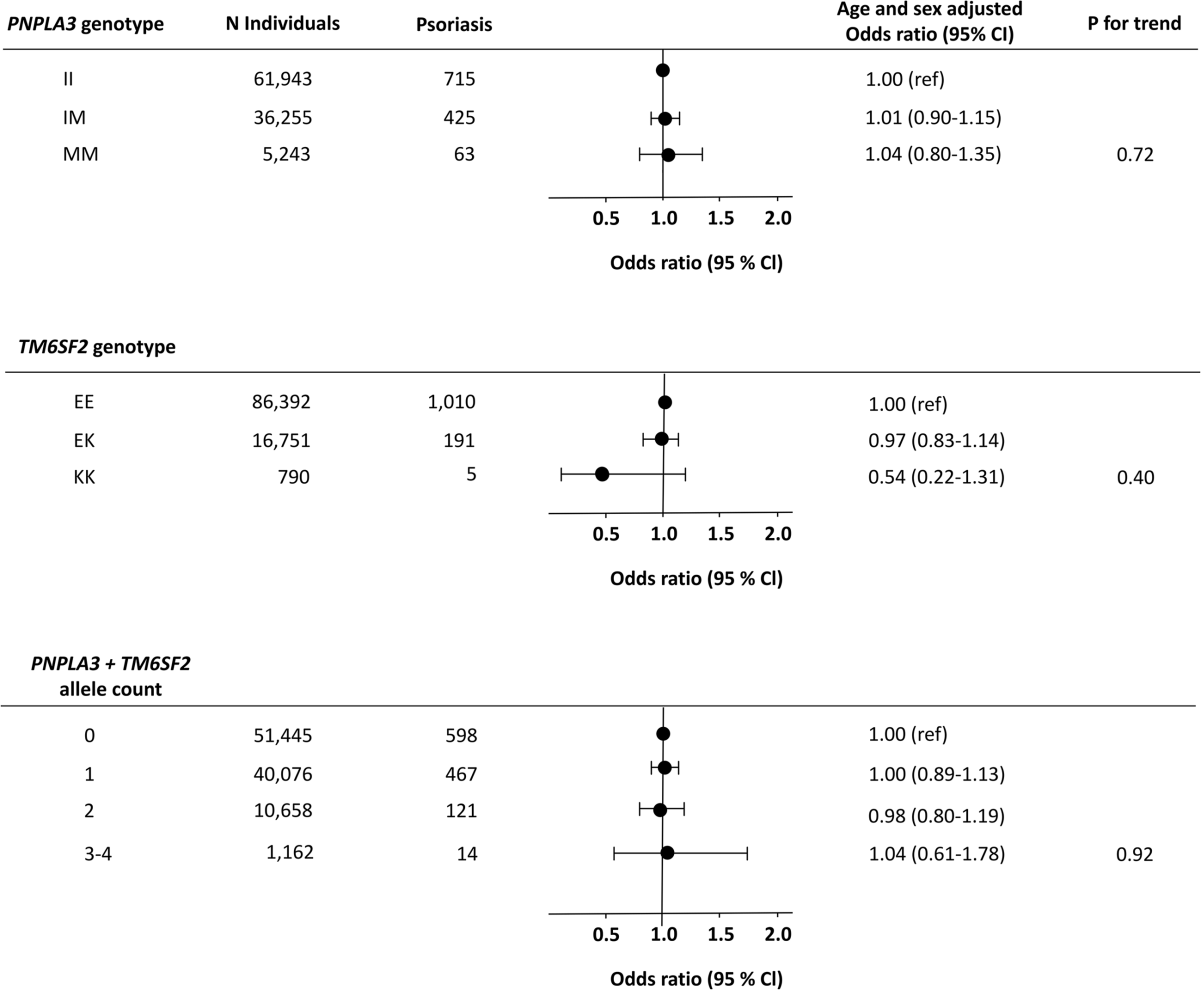
Risk of psoriasis according to NAFLD-promoting genetic variants in individuals from the general population. Upper panel: Risk of psoriasis according to the genotype *PNPLA3*. Middle panel: Risk of psoriasis according to the genotype *TM6SF2*. Lower panel: Risk of psoriasis according to the combined allele count. Analyses were adjusted for age and sex. N, number; NAFLD, non-alcoholic fatty liver disease.

However, the genetic variants were not associated with increased risk of psoriasis (**Figure 4**). For *PNPLA3*, the age and sex adjusted odds ratio of psoriasis was 1.01 (0.90-1.15) in IM-heterozygotes and 1.04 (0.80-1.35) in MM-homozygotes compared to non-carriers (**Figure 4** upper panel). For *TM6SF2*, the age and sex adjusted odds ratio of psoriasis was 0.97 (0.83-1.14) in EK-heterozygotes and 0.54 (0.22-1.31) in KK-homozygotes compared to non-carriers (**Figure 4** middle panel). Also, when using the total allele count, we found no association between the NAFLD-promoting alleles and psoriasis (**Figure 4** lower panel). In sensitivity analyses, similar results were seen using the more restrictive definition of psoriasis (compare **Figure 4** with **Supplementary Figure S7** **)** and when stratifying according to potential confounders including sex, obesity, systemic immune-inflammation index, triglycerides, and type 2 diabetes mellitus (**Supplementary Figure S8** **).** We found no interaction between the included confounders and NAFLD-promoting risk alleles on the risk of psoriasis.

## Discussion

**Figure 5:**
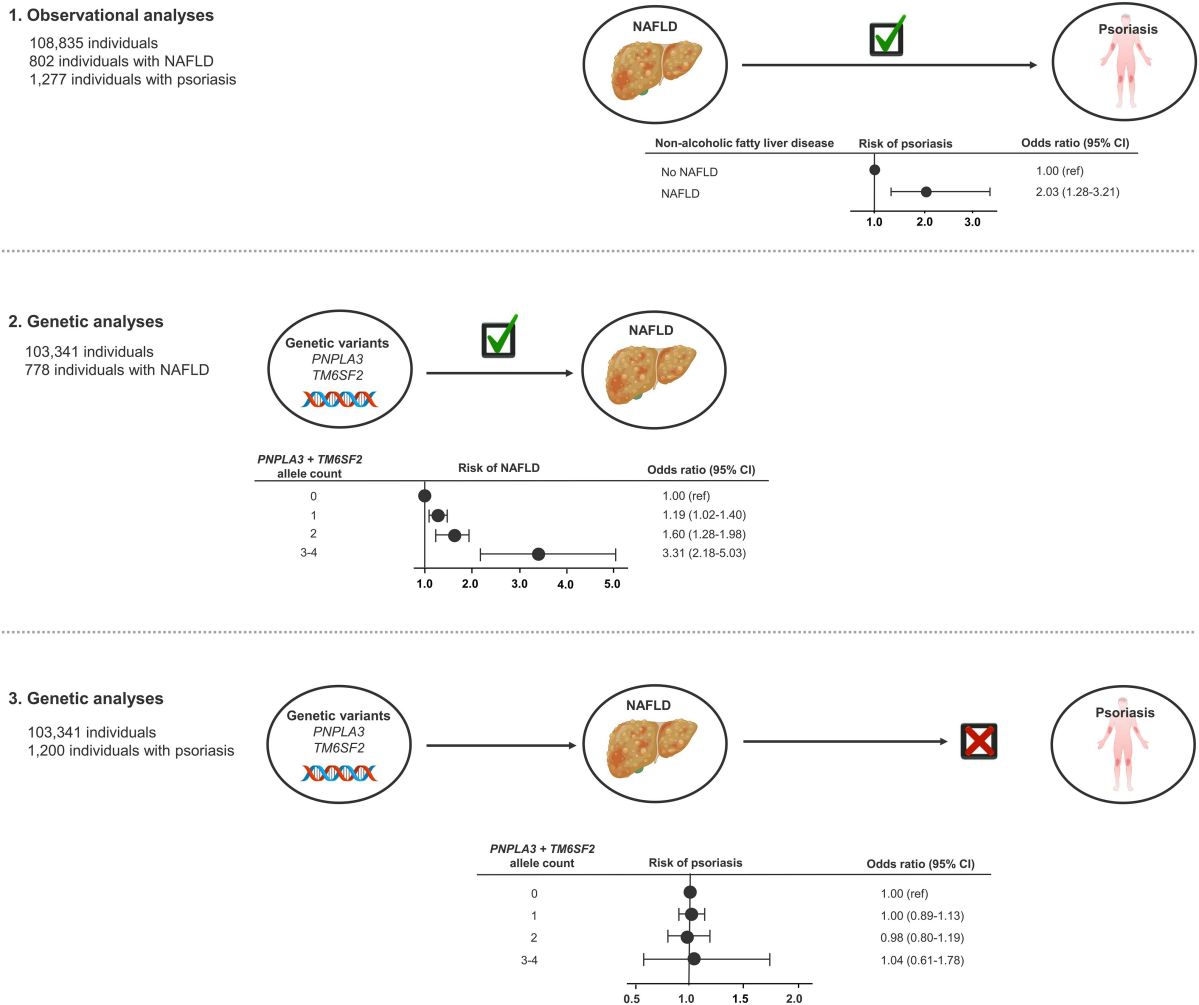
Take-home figure: Observationally, NAFLD was associated with psoriasis (1). The NAFLD-promoting genetic variants were associated with NAFLD (2), but not with increased risk of psoriasis (3). Thus, our study did not find evidence of a causal relationship between NAFLD and psoriasis. NAFLD, non-alcoholic fatty liver disease.

In this MR study of 108,835 individuals from the general population, we found that a diagnosis of NAFLD or high liver fat content is observationally associated with a higher risk of psoriasis. However, genetically determined high liver fat content, a proxy for NAFLD, was not associated with increased risk of psoriasis and thus, we found no evidence of a causal relationship between NAFLD and psoriasis (**Figure 5**).

The mechanisms underlying the observational association between NAFLD and psoriasis are likely complex and multifactorial (11) and could be a result of both confounding and/or reverse causation. NAFLD and psoriasis share several risk factors such as obesity, the metabolic syndrome, and insulin resistance (31–33) and patients with psoriasis are often treated with liver toxic medications (34). In addition, individuals with psoriasis often have high alcohol consumption which also impacts liver function (35). Patients with psoriasis have higher risk of NAFLD (6, 7), however, studies on risk of psoriasis in individuals with NAFLD are scarce. Importantly, circulating pro-inflammatory cytokines associated with both psoriasis and NAFLD drive a chronic low-grade inflammation that could lead to progression of liver damage and worsening of the psoriatic skin (11, 12). Evidence suggest that this can be further aggravated in obese individuals (11). Thus, NAFLD and psoriasis are linked through both shared risk factors and pathophysiology.

The last decades several observational studies have investigated the association between psoriasis and NAFLD (6, 7). In the present study, we find a higher risk of psoriasis in individuals with NAFLD and increased liver fat content, however, confounding and reverse causation are inherent limitation in observational studies. To avoid this, we also used the MR approach. This approach, described as the “natural randomized controlled trial”, is an alternative when randomized controlled trials are unethical or unfeasible and MR studies in medical science, including in the field of psoriasis (36–41), are increasing (42, 43). The present MR results are in agreement with a recent two-sample bidirectional MR study by Zhao et al., investigating causal directions of several comorbidities with psoriasis and psoriasis arthritis (44) without finding a causal association between NAFLD and psoriasis. Furthermore, Zhao and his colleagues did not find evidence of psoriasis being a causal risk factor for NAFLD indicating that the association between NAFLD and psoriasis could be a result of confounding factors.

Strengths of the present study include the large sample size with more than 100,000 individuals, use of the unique Danish health registries, and the comprehensive amount of data on each participant including BMI, biochemistry, alcohol consumption, and genetic analyses. However, potential limitations should be mentioned. As we studied individuals of Danish descent, we potentially reduce the generalizability of our findings although we also minimize the risk of population stratification. The genetic instrument consists of only 2 genotypes, but it is unlikely that inclusion of more genotypes would have altered our results as 1) it is already a strong instrument for liver fat with an F-value of 143 [>10 is considered adequate strength (13)] and 2) the overall causal analysis strongly suggests a non-association (p=0.92). Another limitation of our study is the identification of individuals with NAFLD and psoriasis. We used ICD-8 and ICD-10 codes to identify individuals with hospital-diagnosed psoriasis, which mainly applies to patients with moderate to severe psoriasis (20). Consequently, individuals with mild psoriasis are not included in our psoriasis population, however, this would tend to bias our observational results toward the null hypothesis and thus cannot explain our observational findings. Using ICD-8 and ICD-10 codes to identify individuals with NAFLD, we likely underestimate the prevalence of the disease. However, until recently, NAFLD was not widely recognized clinically, which could be the reason for the low number of diagnoses. Nevertheless, the inclusion of both liver fat measured on CT-scans and the calculated fatty liver index (26) in the present study further strengthens the observational association between NAFLD and psoriasis. Most importantly, an underestimation of the modifiable exposure (in this case NAFLD) does not impact the results in a MR study. Lastly, we do not have access to pharmacological data on participants and therefore cannot take this into account in the present study.

In conclusion, in this Mendelian randomization study we found that high liver fat content or a diagnosis of NAFLD was observationally associated with higher risk of psoriasis. However, we did not find evidence of a causal relationship between NAFLD and psoriasis.

## Data availability statement

The datasets presented in this article are not readily available because data from the Copenhagen General Population Study are subject to protection from the national Danish Data Protection Agency and we are not allowed to share the data ourselves. However, interested researchers can contact members of the steering committee to request data access. Additional data are available upon request and requests may be made to the corresponding author. Requests to access the datasets should be directed to charlotte.sigrid.erika.naeslund.koch@regionh.dk.

## Ethics statement

The studies involving human participants were reviewed and approved by The National Committee on Health Research Ethics (H-KF-01-144/01). The patients/participants provided their written informed consent to participate in this study.

## Author contributions

All authors designed the study. CN-K and SV-K did the statistical analyses. CN-K wrote the first draft of the manuscript and created figures and tables. All authors contributed to the article and approved the submitted version.

## Funding

This study was funded by Krista and Viggo Petersen´s Foundation, Kgl Hofbundtmager Aage Bang Foundation, the Danish National Psoriasis Foundation and Herlev and Gentofte Hospital research fund.

## Acknowledgments

The authors are grateful for the important contributions of the staff and participants of the Copenhagen General Population Study.

## Conflict of interest

CN-K has served as an investigator for Galderma, Abbvie, LEO Pharma, and CSL Behring. LS has received research funding from Novartis, Bristol-Myers Squibb, AbbVie, Janssen Pharmaceuticals, the Danish National Psoriasis Foundation, the LEO Foundation and the Kgl Hofbundtmager Aage Bang Foundation and honoraria as consultant and/or speaker for AbbVie, Eli Lilly, Novartis, Pfizer, and LEO Pharma, Janssen, UCB, Almirall, Bristol-Myers Squibb, and Sanofi. LS has served as an investigator for AbbVie, Pfizer, Sanofi, Janssen, Boehringer Ingelheim, AstraZeneca, Eli Lilly, Novartis, Regeneron, Galderma and LEO Pharma. LG has received research funding from Novo Nordisk, Alexion, Gilead, Vingmed and honoraria as consultant/speaker/expert testimony from Novo Nordisk, Pfizer, Sobi International, and Norgine.

The remaining authors declare that the research was conducted in the absence of any commercial or financial relationships that could be construed as a potential conflict of interest.

## Publisher’s note

All claims expressed in this article are solely those of the authors and do not necessarily represent those of their affiliated organizations, or those of the publisher, the editors and the reviewers. Any product that may be evaluated in this article, or claim that may be made by its manufacturer, is not guaranteed or endorsed by the publisher.
