# Supplementary material for: Non-alcoholic fatty liver disease is not a causal risk factor for psoriasis: A Mendelian randomization study of 108,835 individuals
Source: Front Immunol. 2022 Oct 24;13:1022460. doi: 10.3389/fimmu.2022.1022460 (PMC9638101; doi:10.3389/fimmu.2022.1022460)
Supplement: Supplementary file 1 [file DataSheet_1.docx]

Supplementary Material

# Supplementary Data

The following tables and figures are attached in this document:
- Table S1
- Figure S1
- Figure S2
- Figure S3
- Figure S4
- Figure S5
- Figure S6
- Figure S7
- Figure S8

# Supplementary Table 1

Baseline characteristics of the 108,835 participants included from the Copenhagen General Population Study.

|  | **Individuals with NAFLD** N = 802 | **Individuals without NAFLD** N = 108,033 | **P-value** |
| --- | --- | --- | --- |
| Age (years), median (IQR) | 61 (52-68) | 58 (48-67) | 3 ‧ 10^-6^ |
| Women, N (%) | 486 (61) | 59,413 (55) | 0.001 |
| BMI (kg/m^2^), median (IQR) | 28 (25-31) | 26 (23-28) | 2 ‧ 10^-30^ |
| Waist circumference (cm), median (IQR) | 95 (85-104) | 89 (80-98) | 4 ‧ 10^-26^ |
| Waist-to-hip ratio, median (IQR) | 0.91 (0.84-0.97) | 0.88 (0.81-0.94) | 4 ‧ 10^-26^ |
| Low physical activity, N (%) | 76 (9.7) | 6,623 (6.2) | 5 ‧ 10^-5^ |
| Hypertension, N (%) | 409 (55) | 47,688 (48) | 3 ‧ 10^-5^ |
| Dyslipidaemia, N (%) | 572 (72) | 78,755 (73) | 0.42 |
| Triglycerides (mmol/L), median (IQR) | 1.7 (1.1-2.6) | 1.4 (0.96-2.0) | 2 ‧ 10^-20^ |
| Non-fasting glucose (mmol/L), median (IQR) | 5.3 (4.8-6.0) | 5.1 (4.7-5.6) | 1 ‧ 10^-8^ |
| Type 2 diabetes mellitus, N (%) | 105 (13) | 4,448 (4.1) | 9 ‧ 10^-37^ |
| Current smokers, N (%) | 174 (22) | 18,377 (17) | 1 ‧ 10^-4^ |
| Former smokers, N (%) | 341 (43) | 43,934 (41) |  |
| Never smokers, N (%) | 283 (35) | 45,105 (42) |  |
| Alcohol intake (g/week), median (IQR) | 84 (36-180) | 96 (48-180) | 0.02 |

Abbreviations: BMI, body mass index

# Supplementary Figure S1


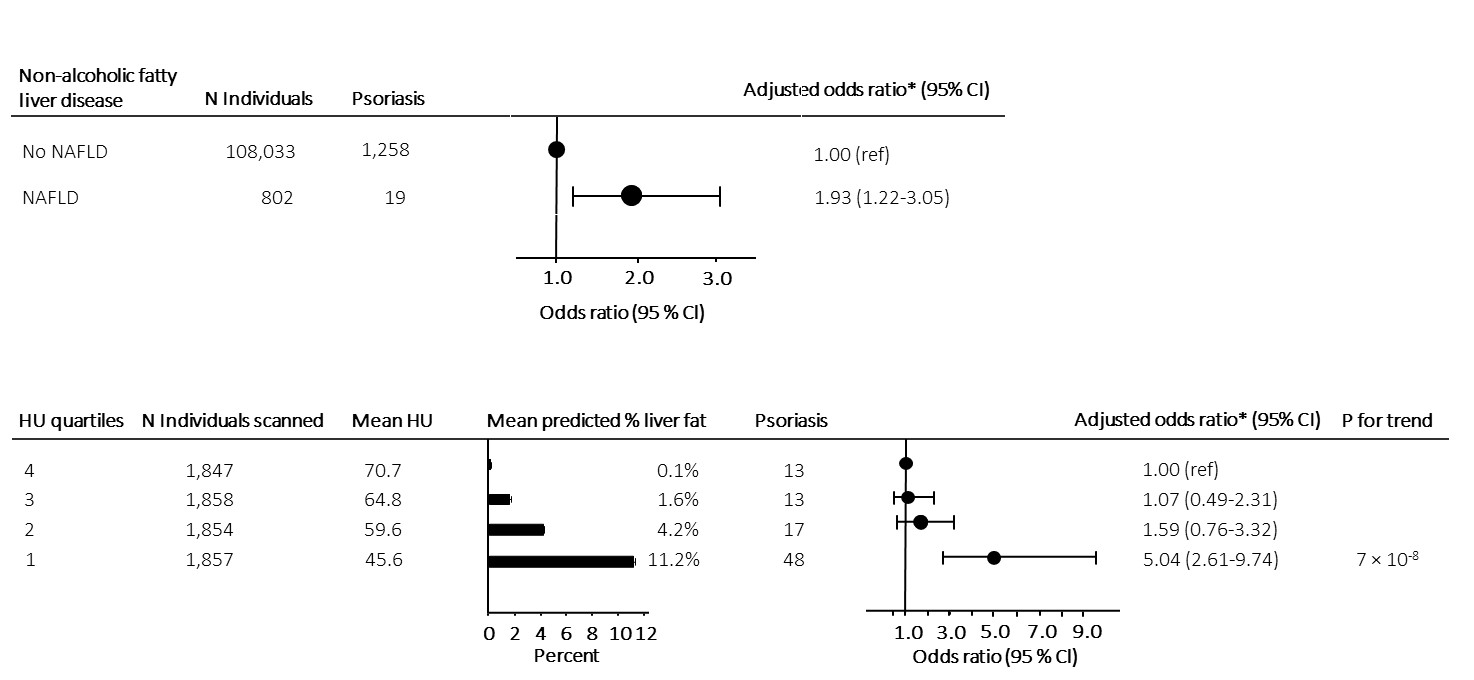


**Supplementary Figure 1.**
Risk of psoriasis according to a diagnosis of NAFLD and liver fat content adjusted for sex, age, triglycerides, dyslipidaemia, and remnant cholesterol.

Upper panel: Risk of psoriasis in individuals with NAFLD compared to individuals without NAFLD from the general population. All cases of NAFLD and psoriasis during follow-up were included in the analysis.

Lower panel: Mean predicted liver fat content and risk of psoriasis in 7,023 individuals with information on liver fat content from CT-scans. Individuals were grouped according to liver fat content in quartiles and risk assessment was done with individuals in the lowest quartile (lowest liver fat content) as reference.

*Analyses were adjusted for sex, age, triglycerides, dyslipidaemia, and remnant cholesterol measured at baseline.

Abbreviations: NAFLD, non-alcoholic fatty liver disease; N, number; HU, Hounsfield units

# Supplementary Figure 2

**
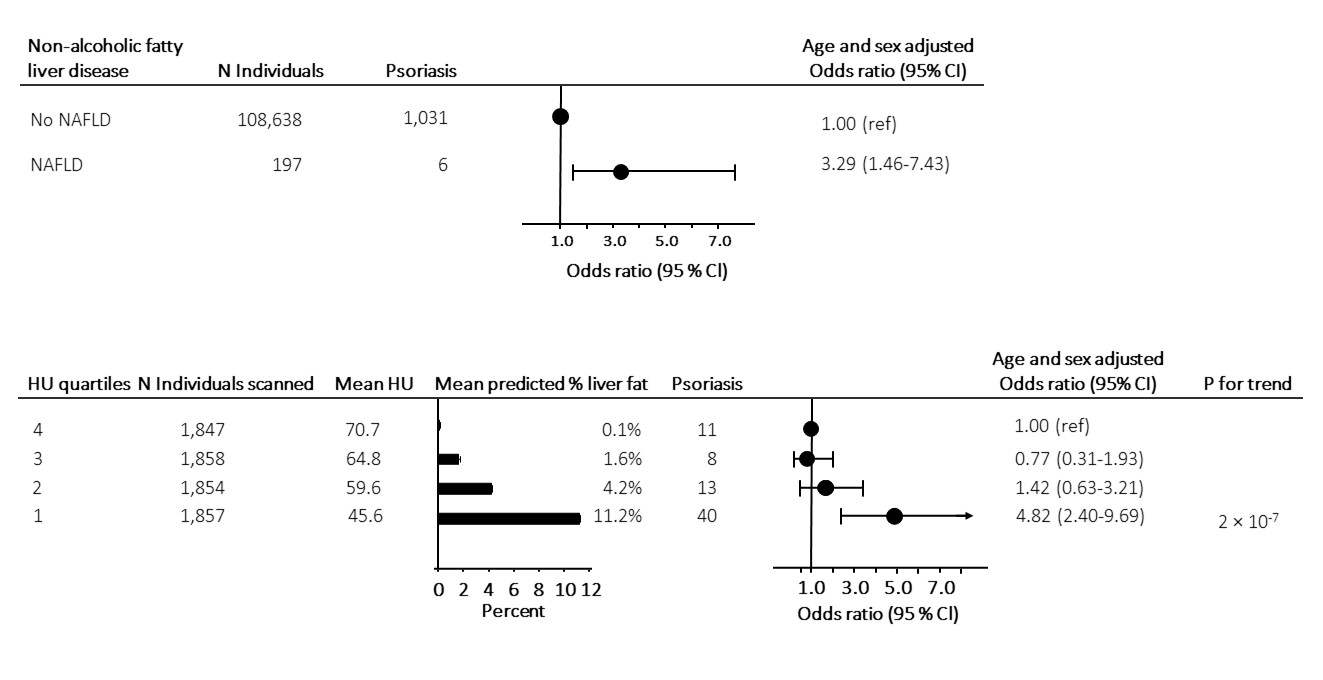
**

**Supplementary Figure 2.**Risk of psoriasis according to a diagnosis of NAFLD and liver fat content using more restrictive diagnostic definitions of NAFLD and psoriasis.

Upper panel: Risk of psoriasis (using ICD-8; 696.19 and ICD-10; L40.0 and L40.9) in individuals with NAFLD (using ICD-8 code; 571.11 and ICD-10; K76.0) compared to individuals without NAFLD from the general population. All cases of NAFLD and psoriasis during follow-up were included in the analysis.

Lower panel: Mean predicted liver fat content and risk of psoriasis (using ICD-8; 696.19 and ICD-10; L40.0 and L40.9) in 7,023 individuals with information on liver fat content from CT-scans. Individuals were grouped according to liver fat content in quartiles and risk assessment was done with individuals in the lowest quartile (lowest liver fat content) as reference.

Analyses were adjusted for sex and age.

Abbreviations: NAFLD, non-alcoholic fatty liver disease; N, number; HU, Hounsfield units

#
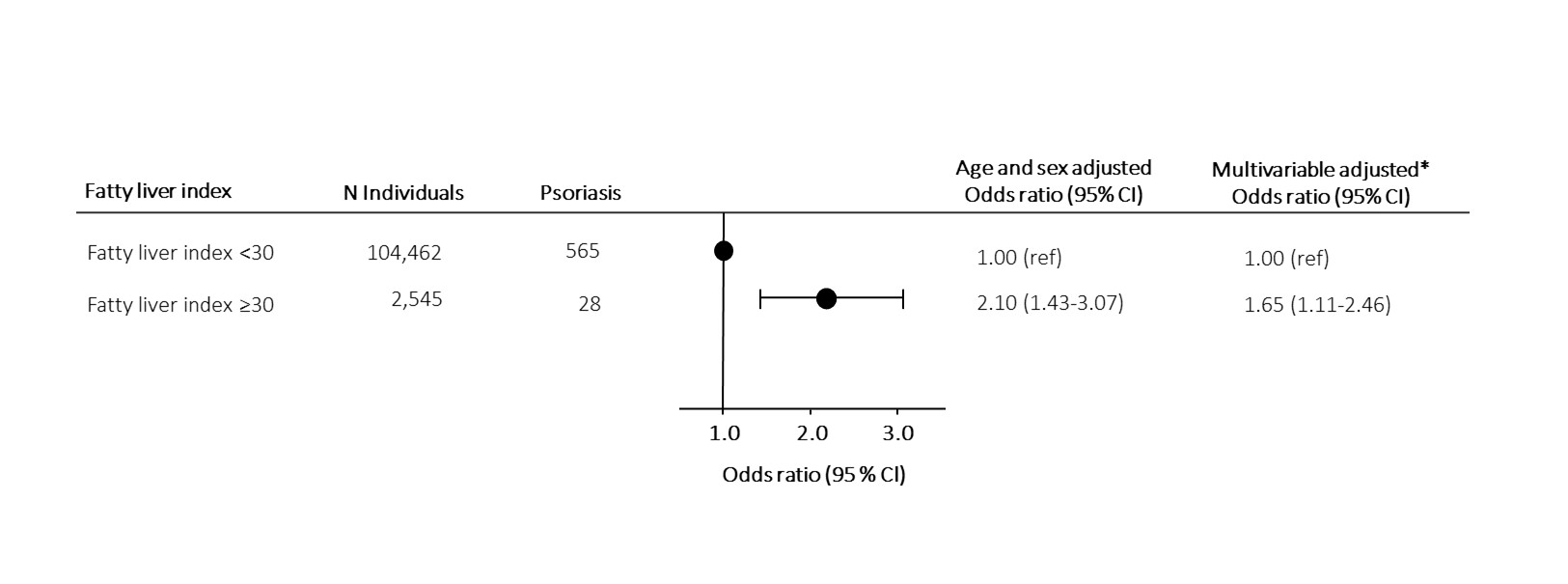
Supplementary Figure 3

**Supplementary Figure 3.**
Risk of psoriasis according to fatty liver index (<30 versus ≥30) measured at baseline.
Only prevalent cases of psoriasis were included in the analysis.

^*^Multivariable adjustments were for hypertension (yes/no), dyslipidaemia (yes/no), smoking (yes/no), excessive alcohol consumption (yes/no), low physical activity (yes/no), type 2 diabetes mellitus (yes/no), and low education (yes/no).

Abbreviations: N, number

# Supplementary Figure 4


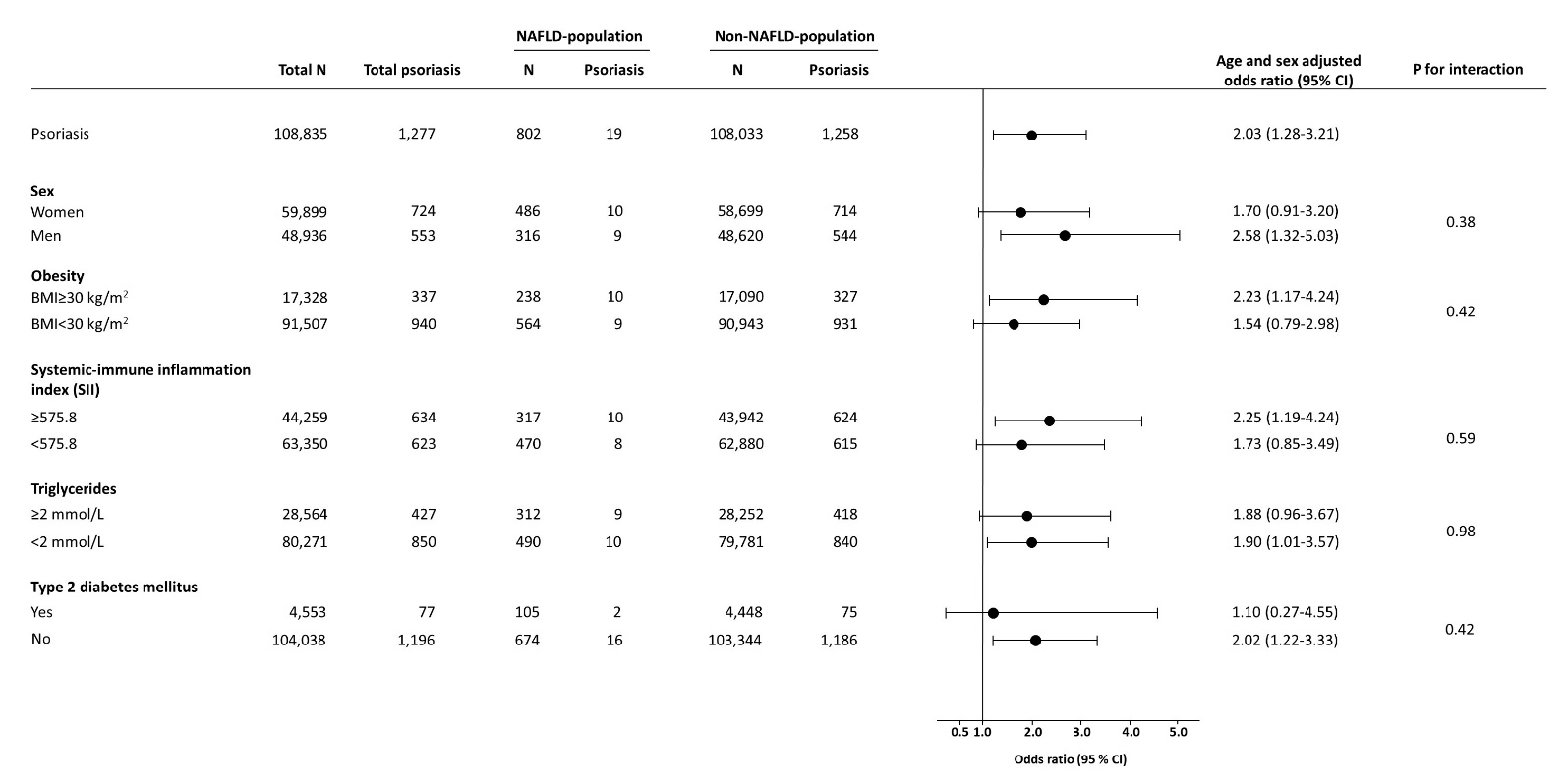


**Supplementary Figure 4.** Risk of psoriasis in individuals with NAFLD compared to individuals without NAFLD stratified according to potential confounders measured at baseline.

Abbreviations: N, number; NAFLD, non-alcoholic fatty liver disease; BMI, body mass index

# Supplementary Figure 5

**
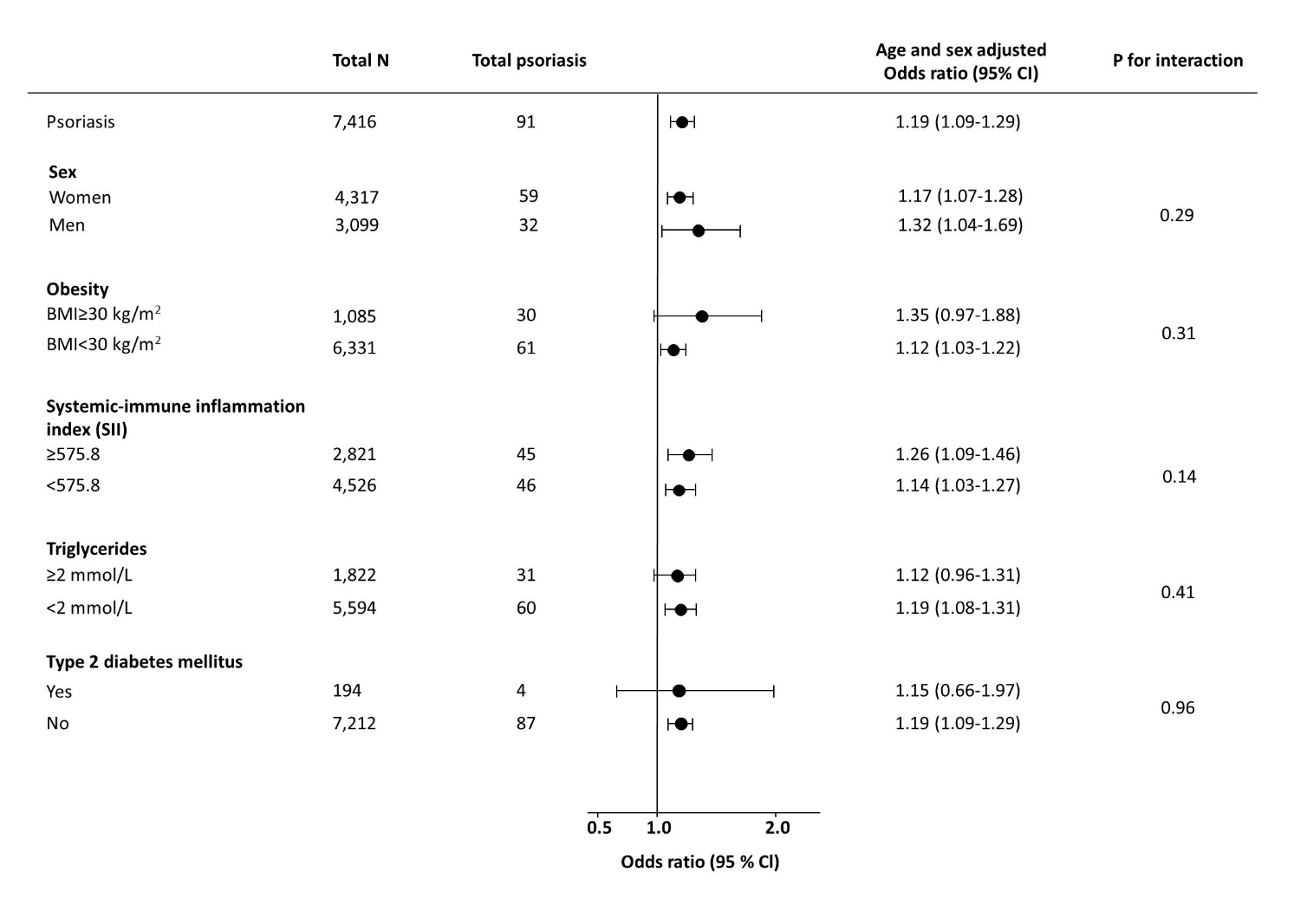
**

**Supplementary Figure 5.** Risk of psoriasis per doubling in liver fat content in 7,416 individuals with information on liver fat content from CT-scans stratified according to potential confounders measured at baseline.

Abbreviations: N, number; NAFLD, non-alcoholic fatty liver disease; BMI, body mass index

# Supplementary Figure 6


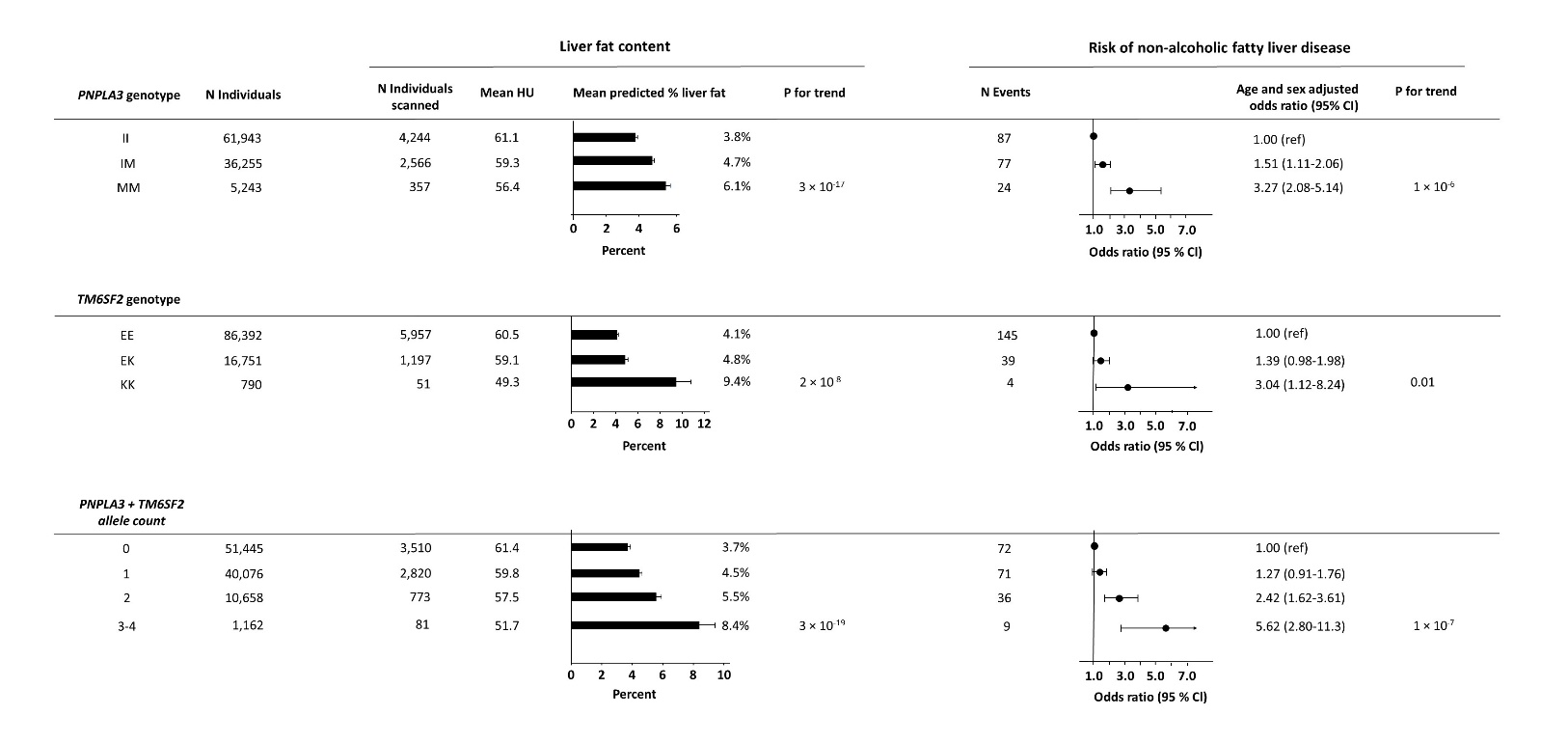


**Supplementary Figure 6.** Liver fat content according to NAFLD-promoting genetic variants in 7,023 individuals and risk of NAFLD (using ICD-8 code; 571.11 and ICD-10; K76.0) according to NAFLD-promoting genetic variants in all individuals.

Upper panel: Liver fat content and risk of NAFLD according to the genotype *PNPLA3*.
Middle panel: Liver fat content and risk of NAFLD according to the genotype *TM6SF2*.
Lower panel: Liver fat content and risk of NAFLD according to the combined allele count.

Analyses were adjusted for age and sex.

Abbreviations: N, number; NAFLD, non-alcoholic fatty liver disease; HU, Hounsfield units

# Supplementary Figure 7


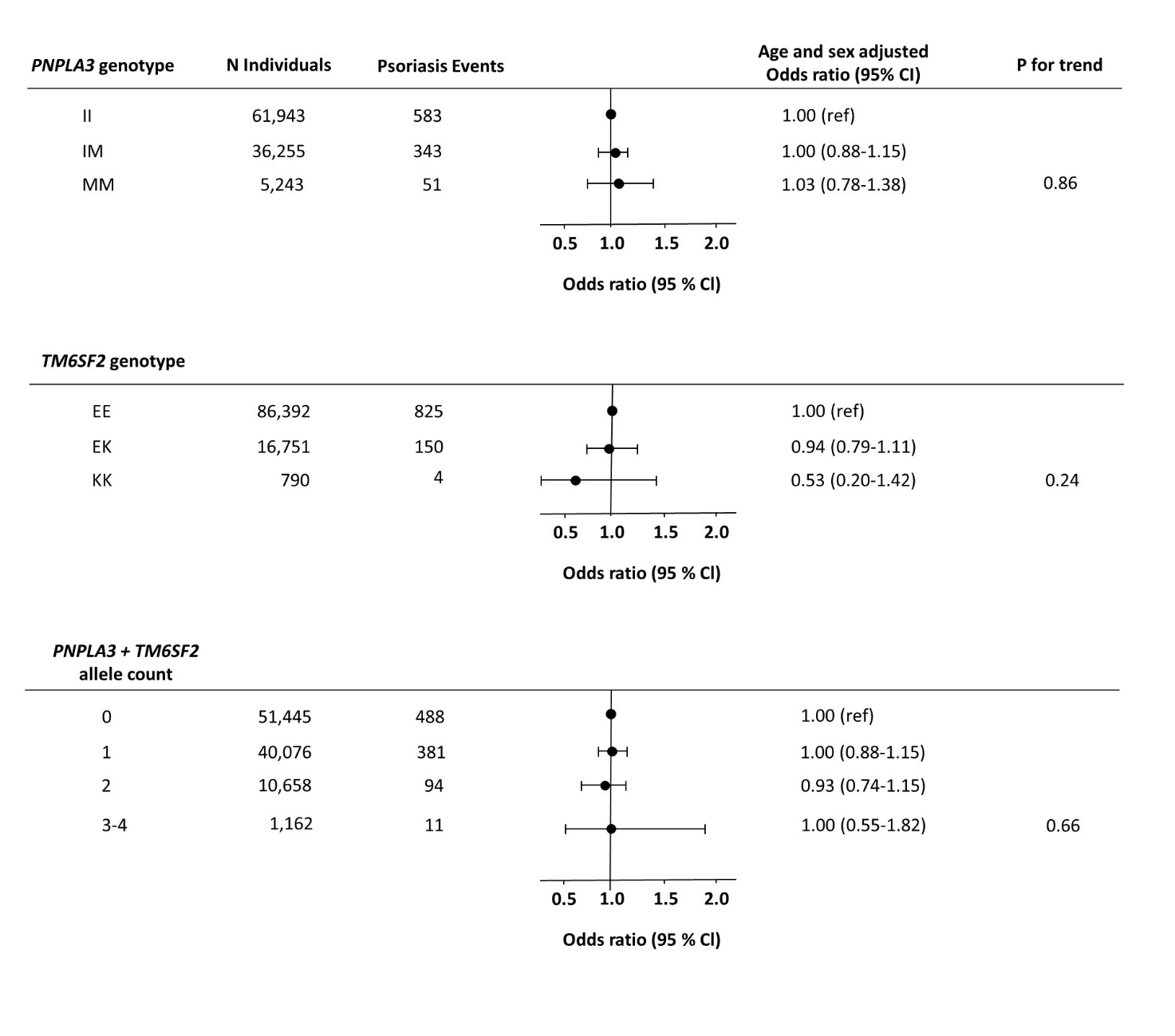


**Supplementary Figure 7.** Risk of psoriasis (using ICD-8; 696.19 and ICD-10; L40.0 and L40.9) according to NAFLD-promoting genetic variants in individuals from the general population.

Upper panel: Risk of psoriasis according to the genotype *PNPLA3*.
Middle panel: Risk of psoriasis according to the genotype *TM6SF2*.
Lower panel: Risk of psoriasis according to the combined allele count.

Analyses were adjusted for age and sex.

Abbreviations: N, number; NAFLD, non-alcoholic fatty liver disease

# Supplementary Figure 8


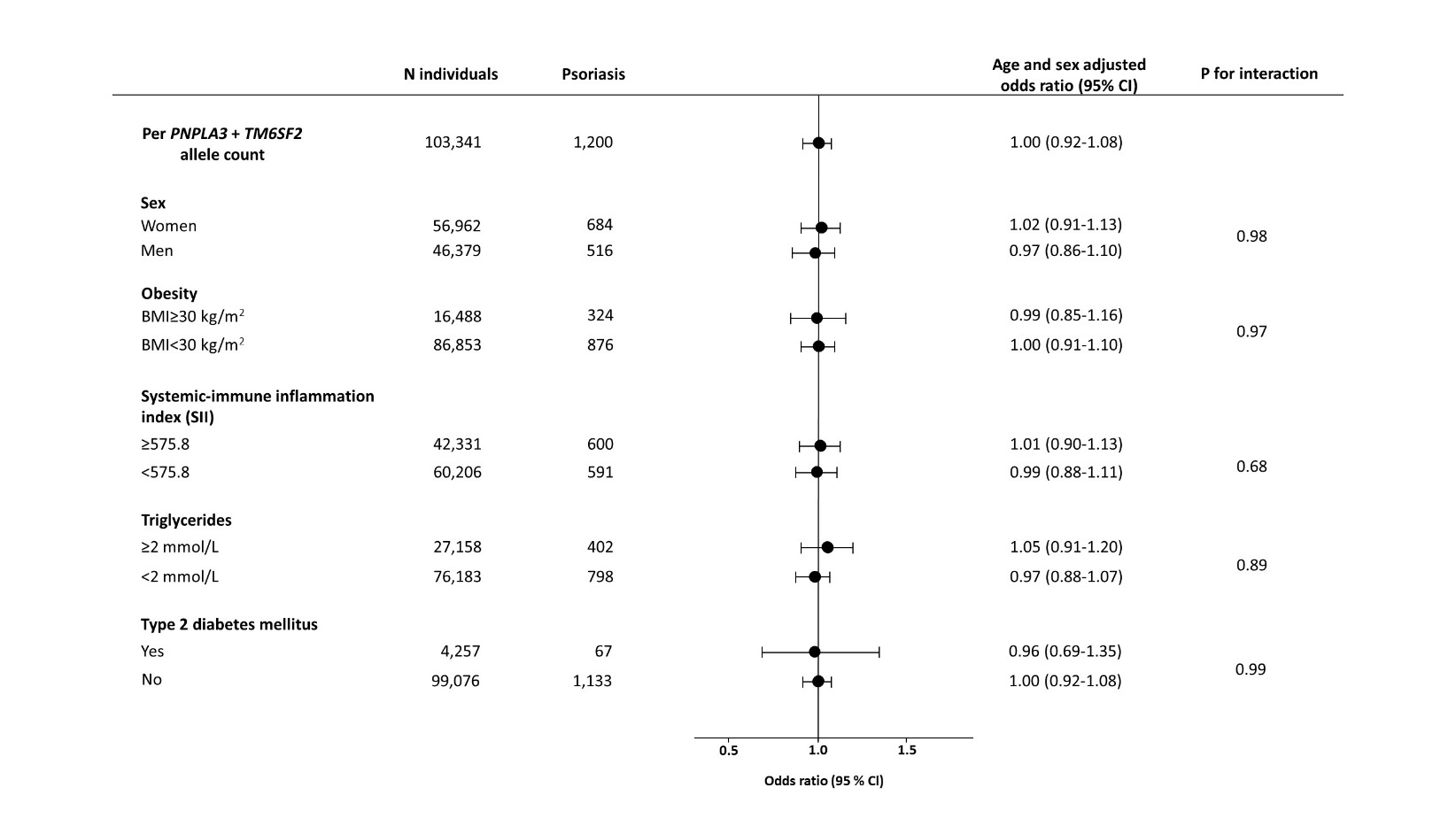


**Supplementary Figure 8.** Risk of psoriasis per one unit increase in the combined NAFLD-promoting risk allele count stratified according to potential confounders measured at baseline.

Analyses were adjusted for age and sex.

Abbreviations: N, number; NAFLD, non-alcoholic fatty liver disease
